# Supplementary figures and images for: TuBA: Tunable biclustering algorithm reveals clinically relevant tumor transcriptional profiles in breast cancer
Source: Gigascience. 2019 Jun 18;8(6):giz064. doi: 10.1093/gigascience/giz064 (PMC6582332; doi:10.1093/gigascience/giz064)

Fig. S1

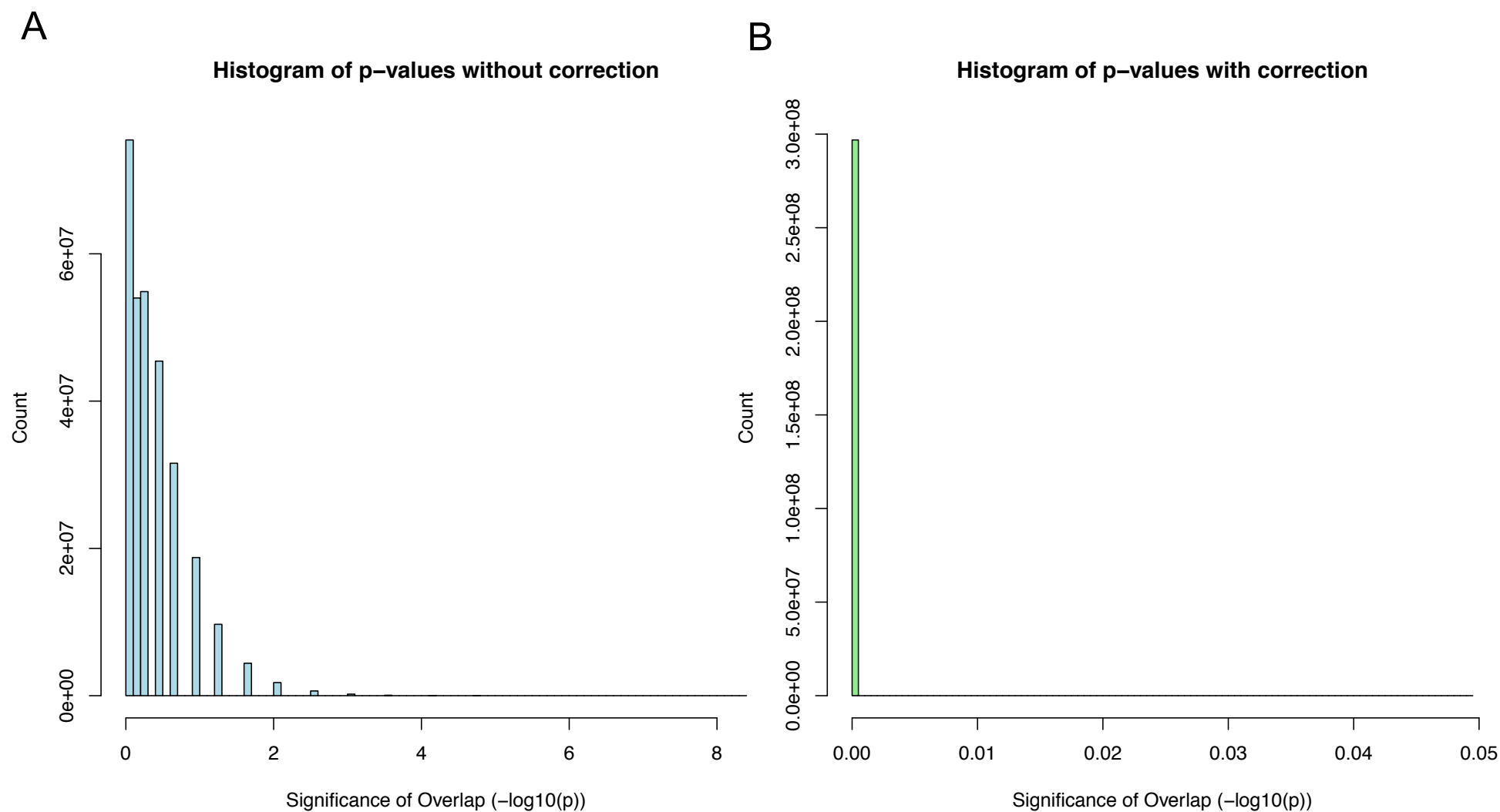

Fig. S2

A

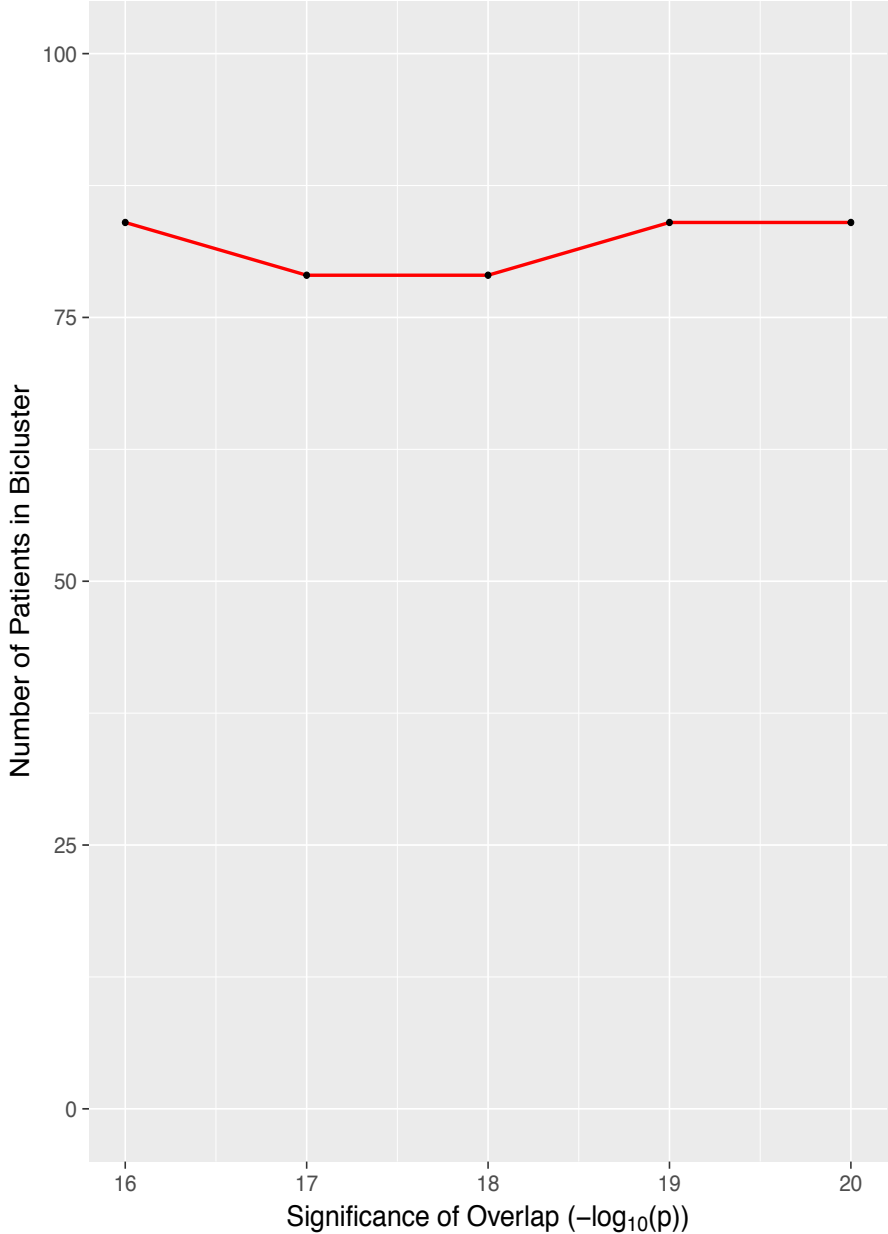

B

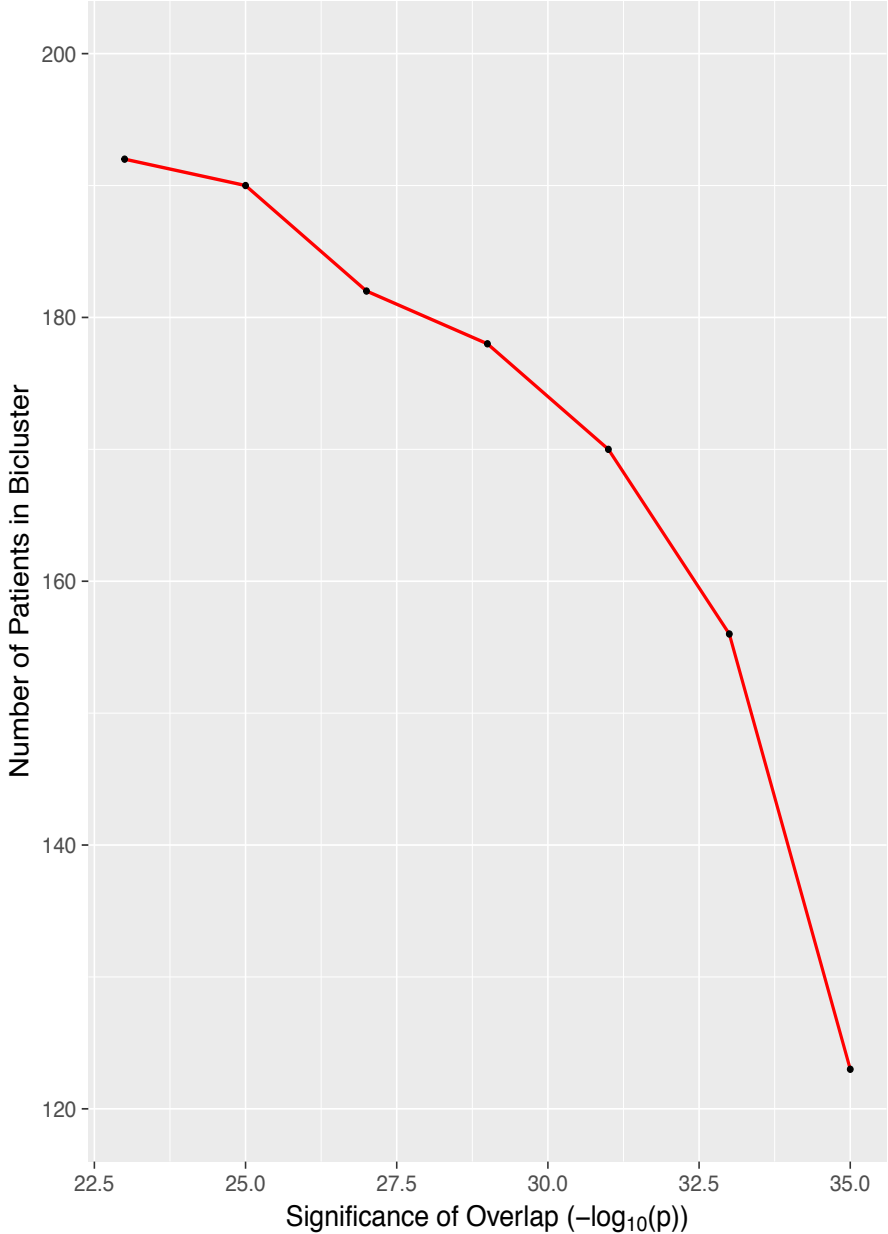

Fig. S3

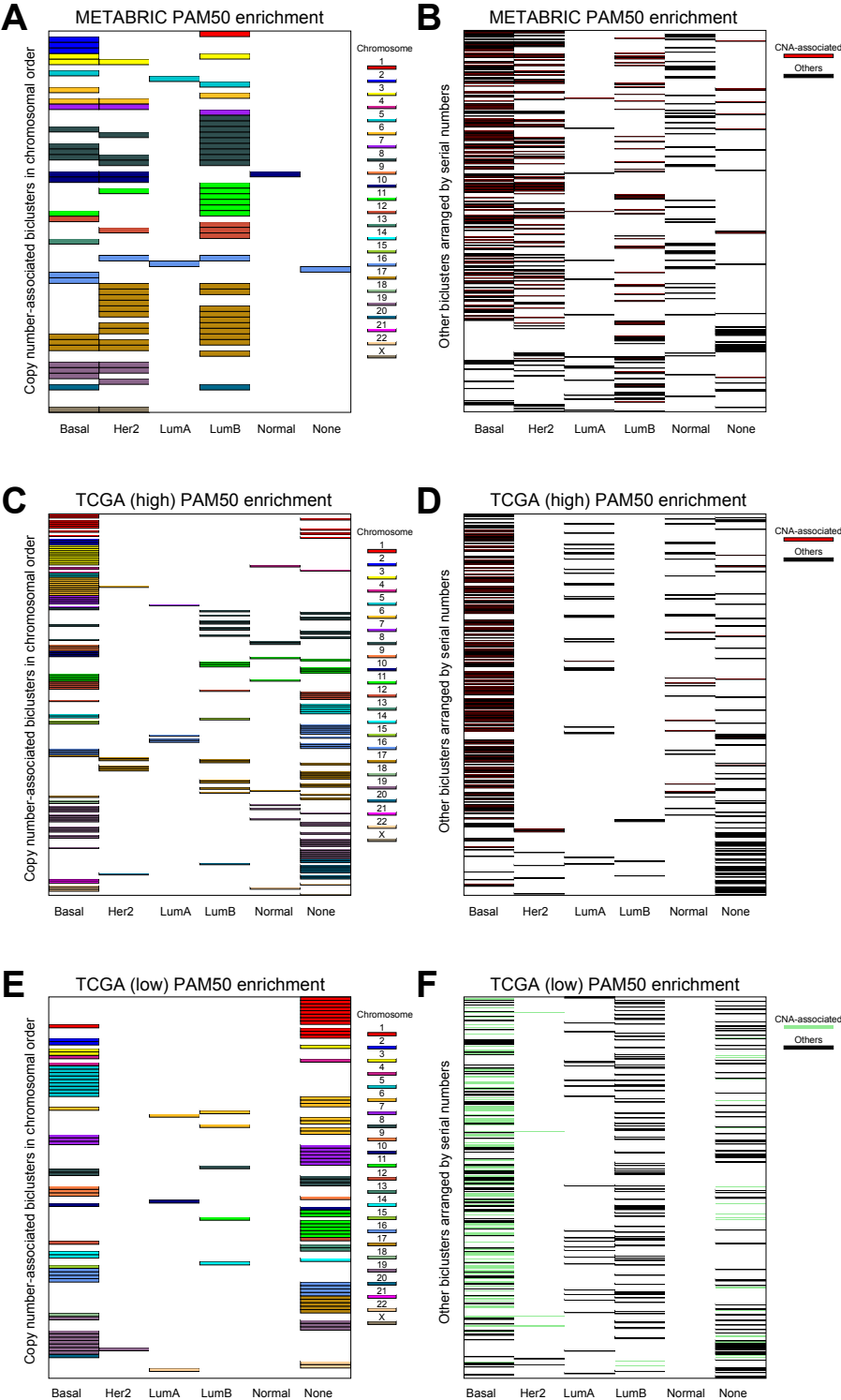

Fig. S4

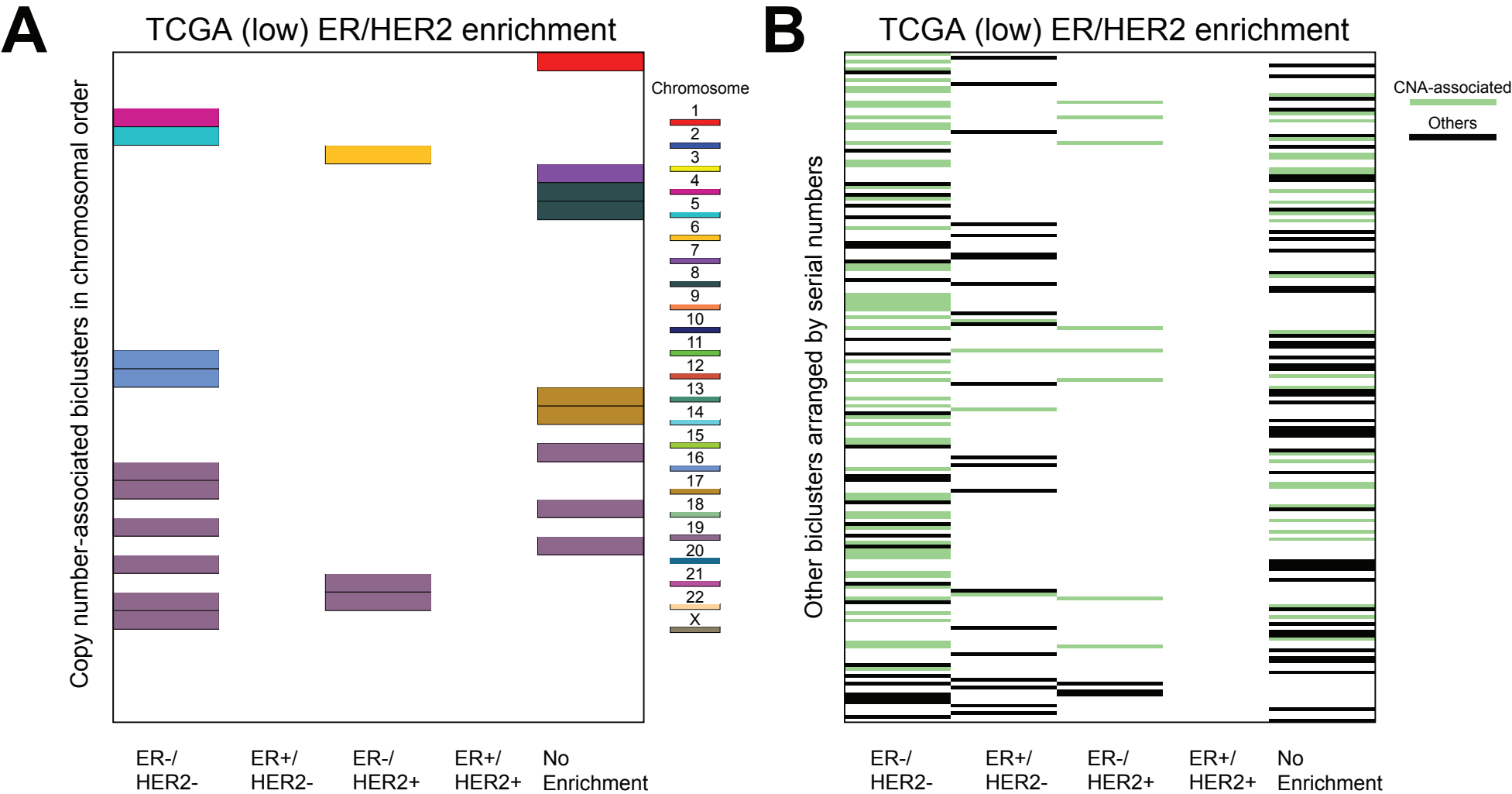

Fig. S5

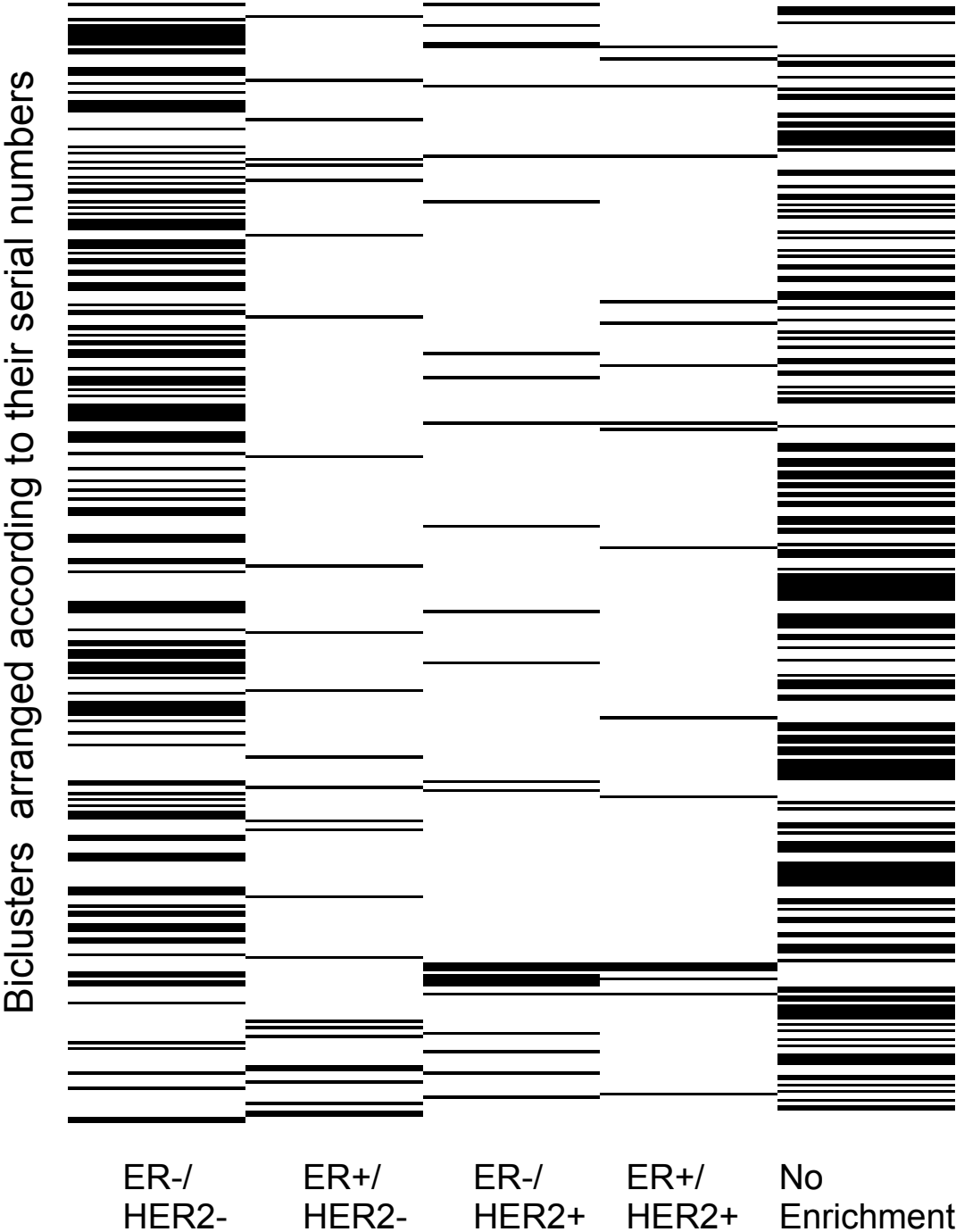

Fig. S6A

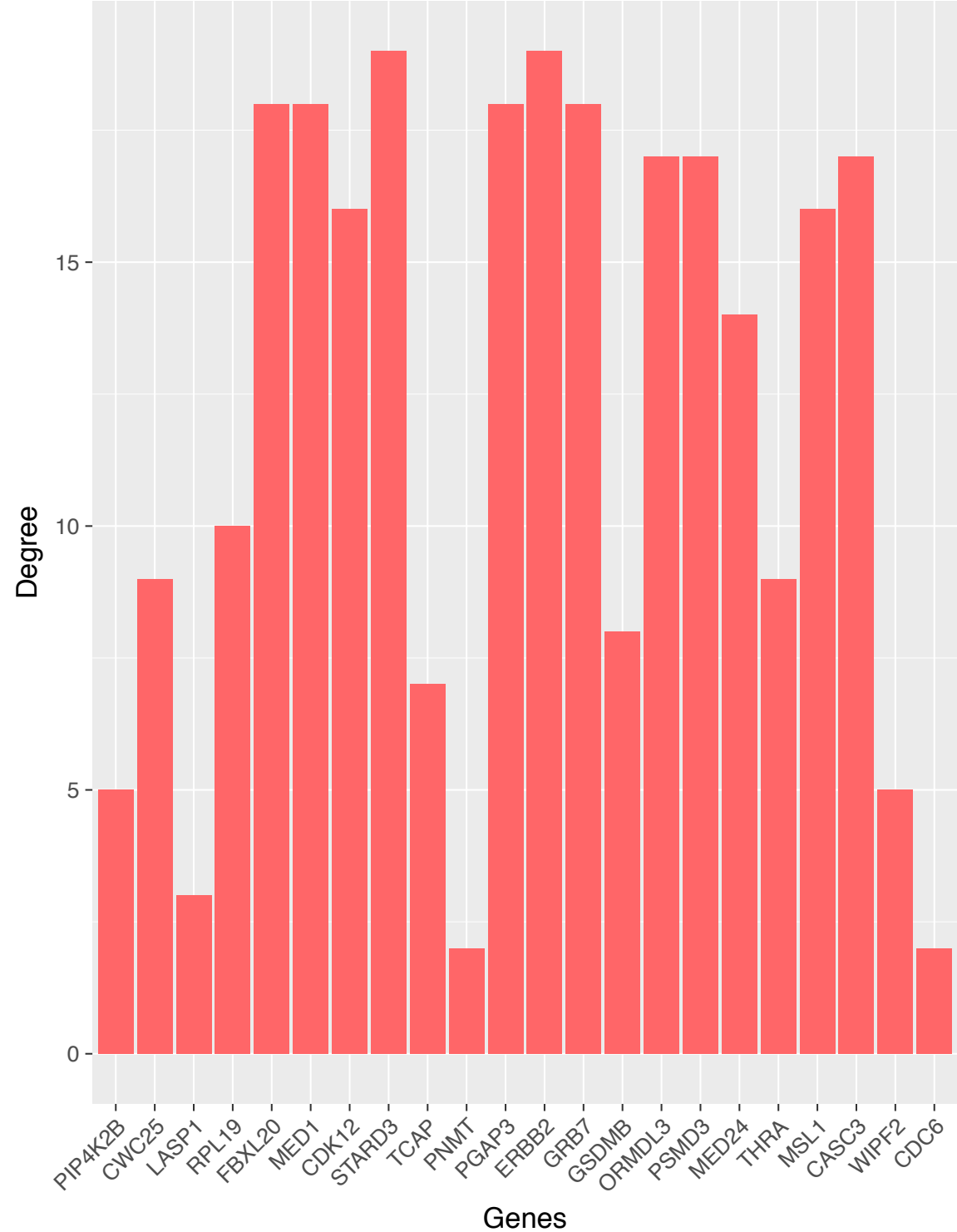

Fig. S6B

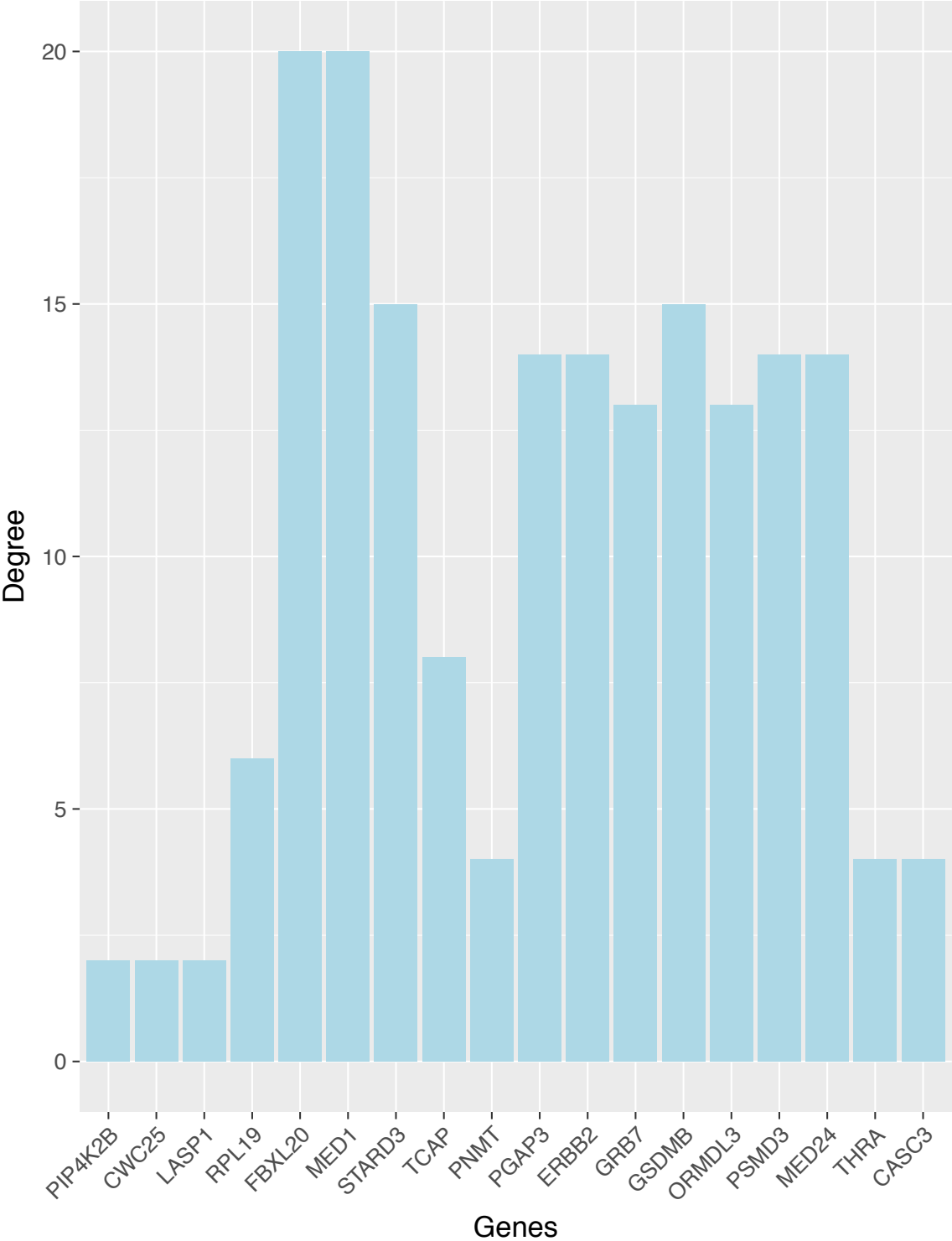

Fig. S6C

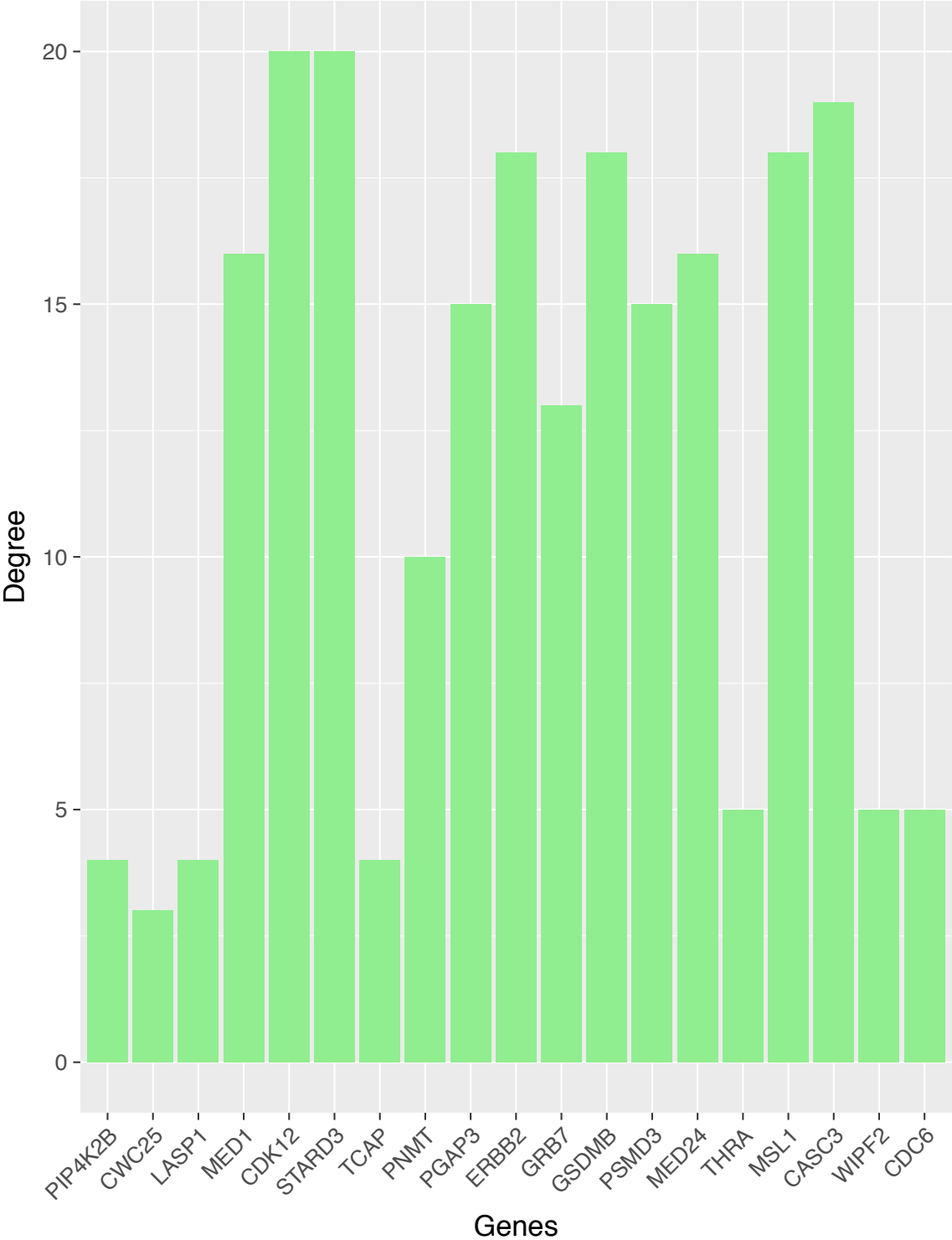

Fig. S7A

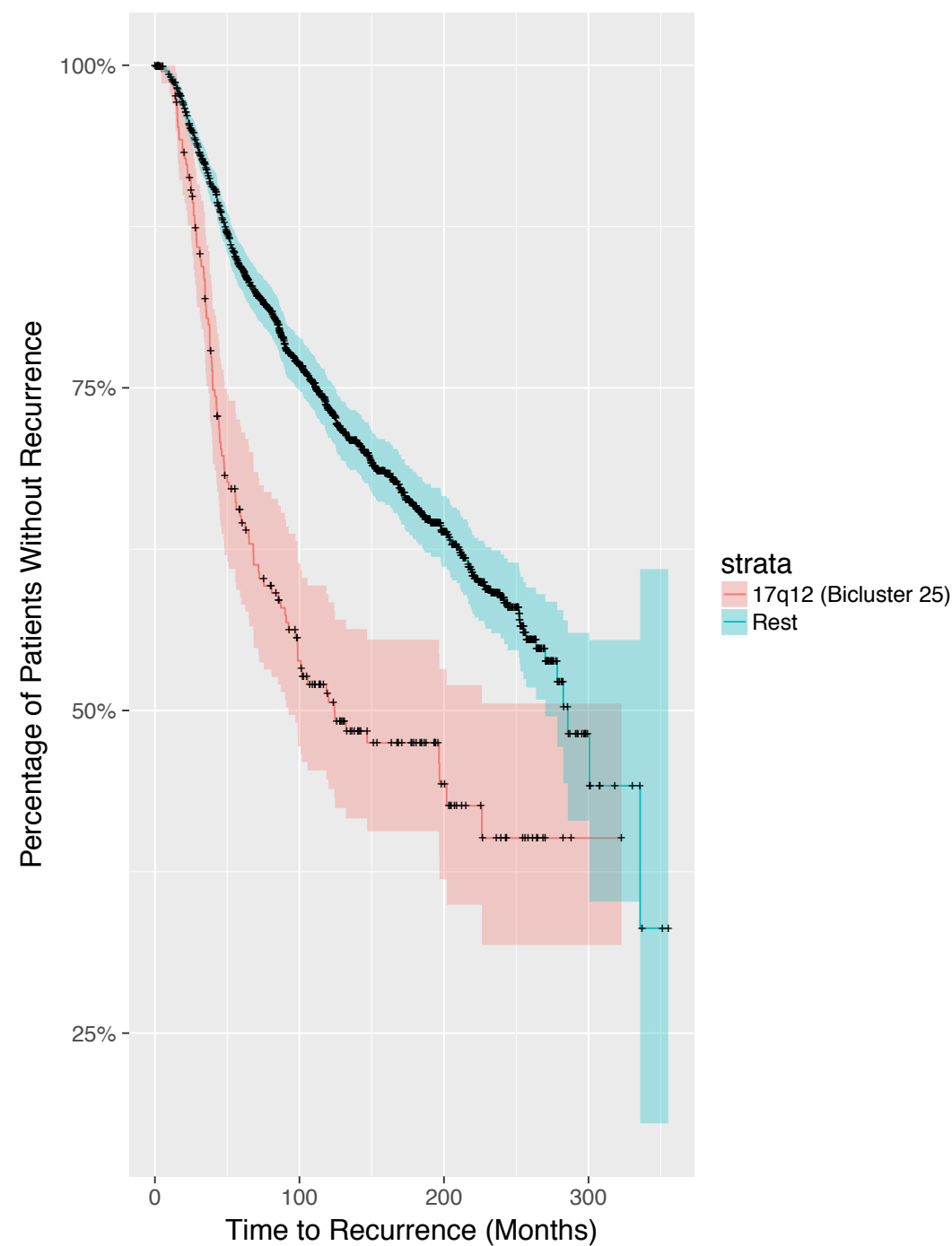

Fig. S7B

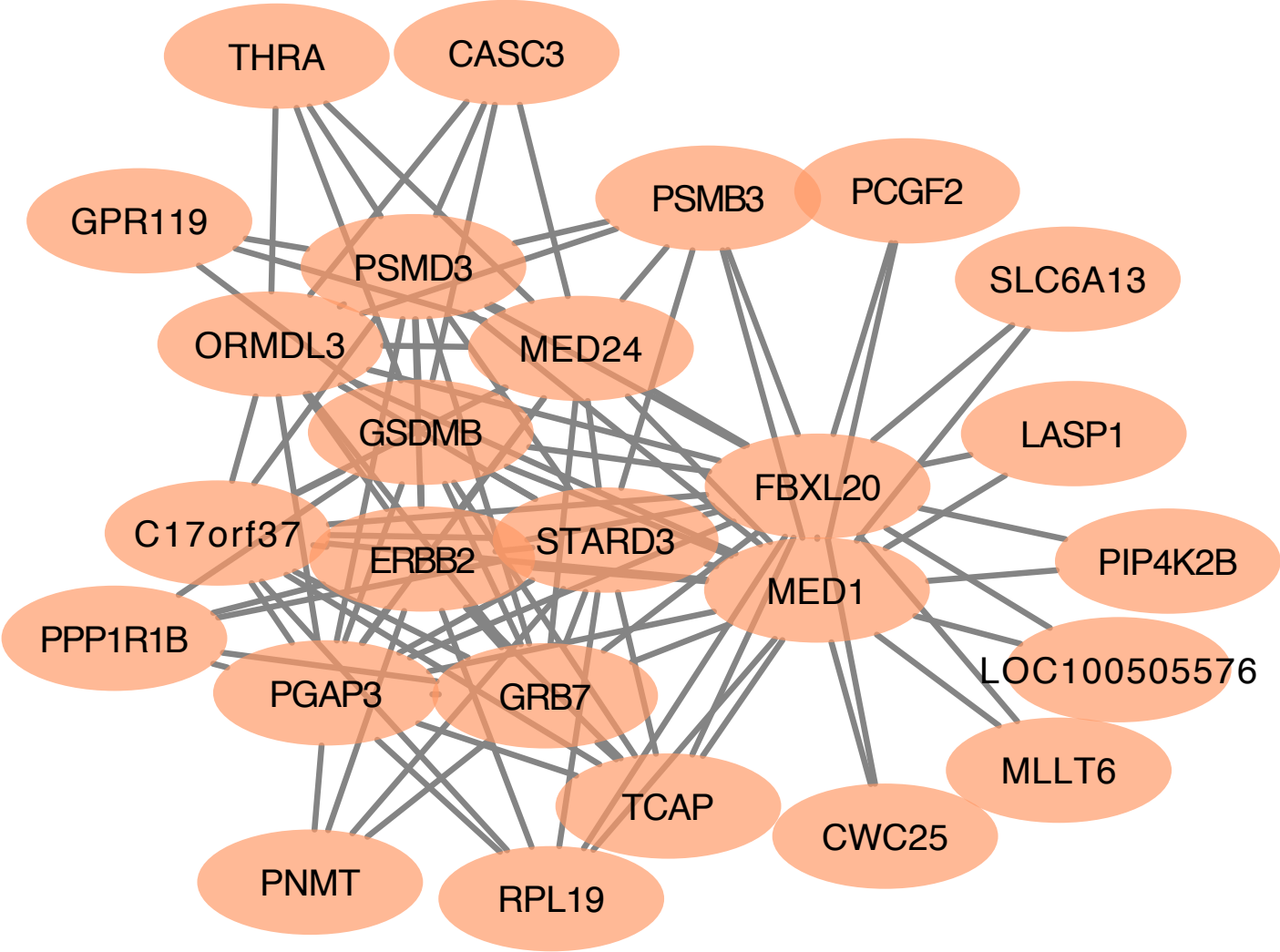

Fig. S8A

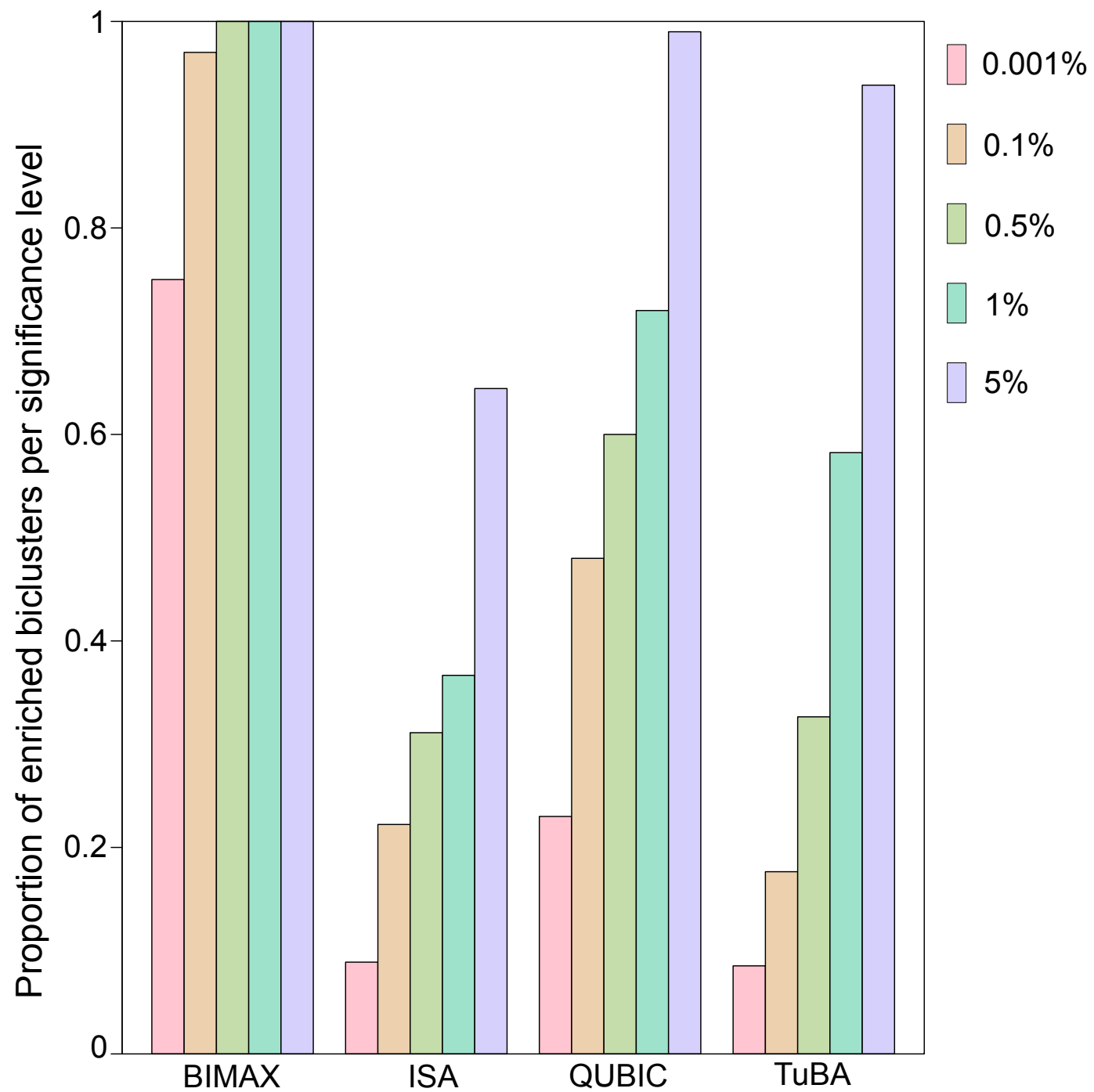

Fig. S8B

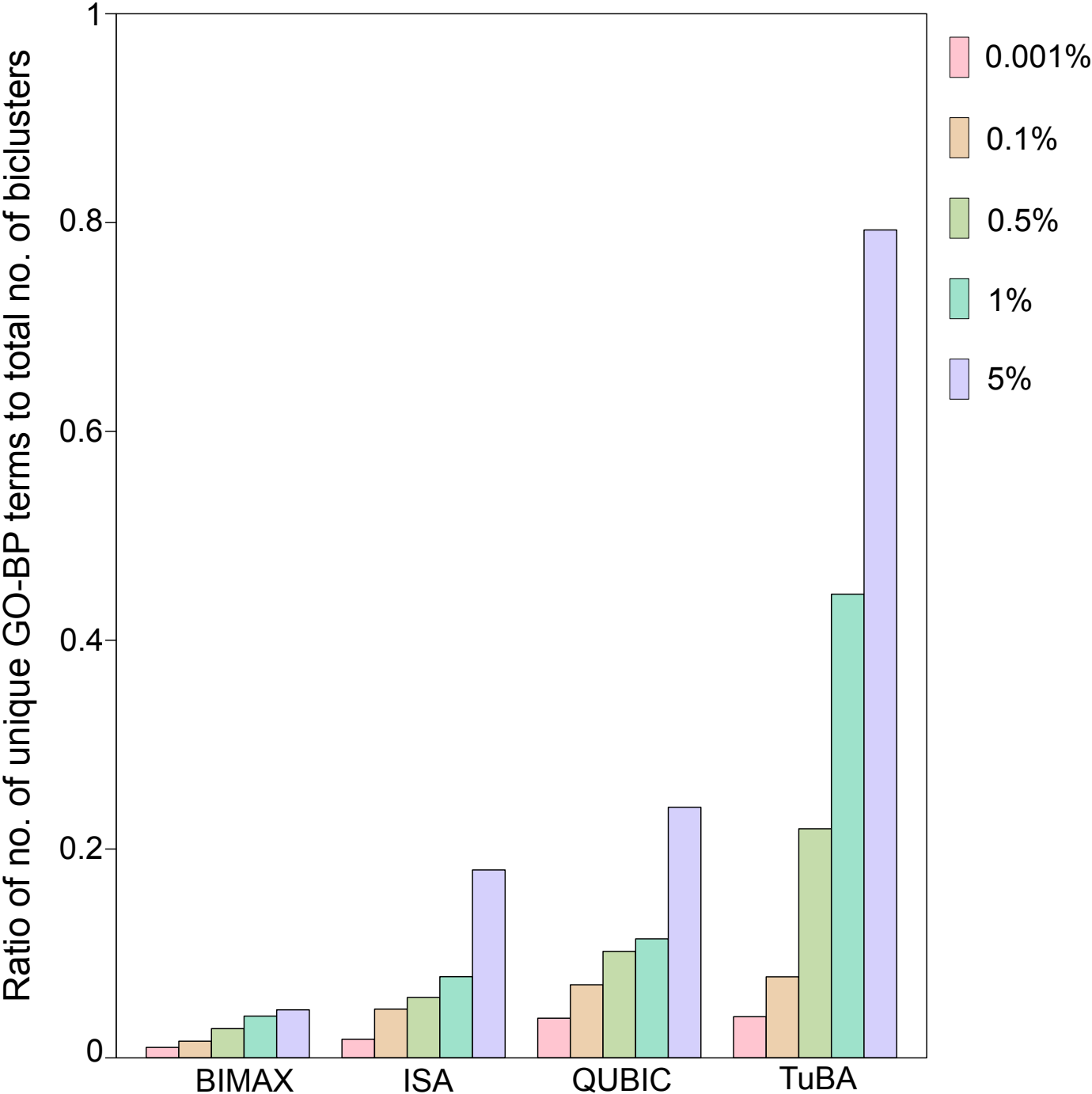

Fig. S9A

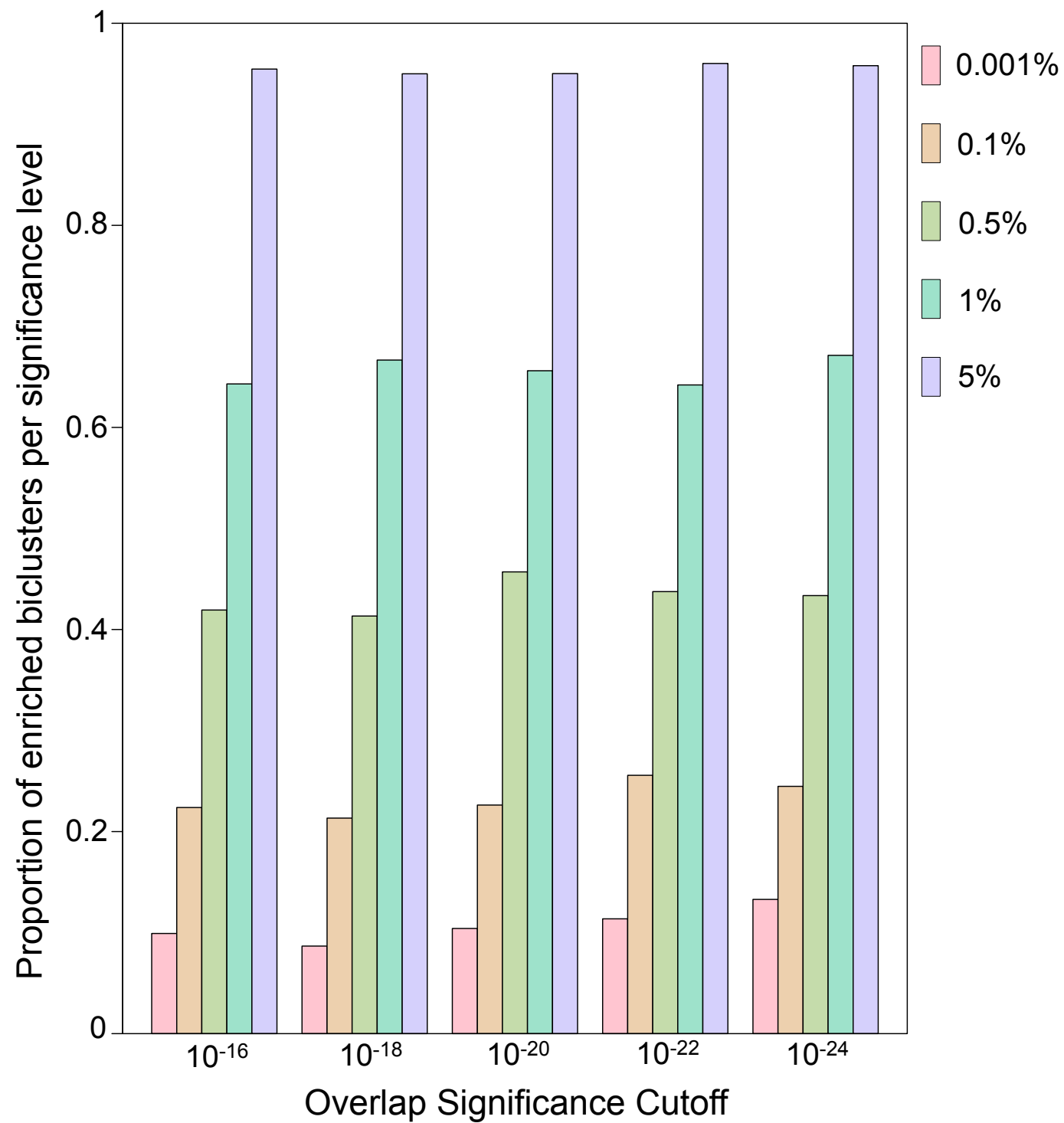

Fig. S9B

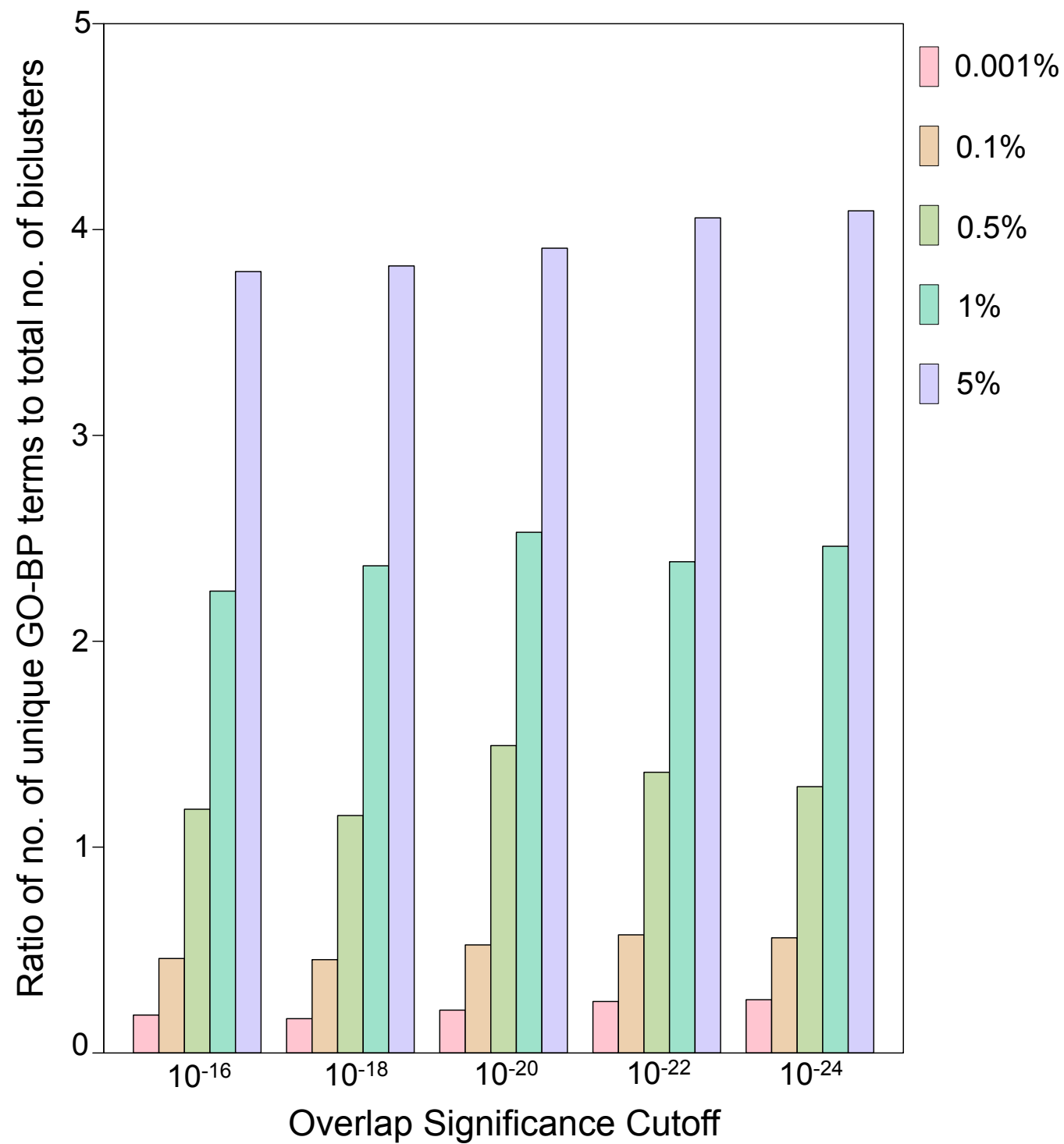

Fig. S10

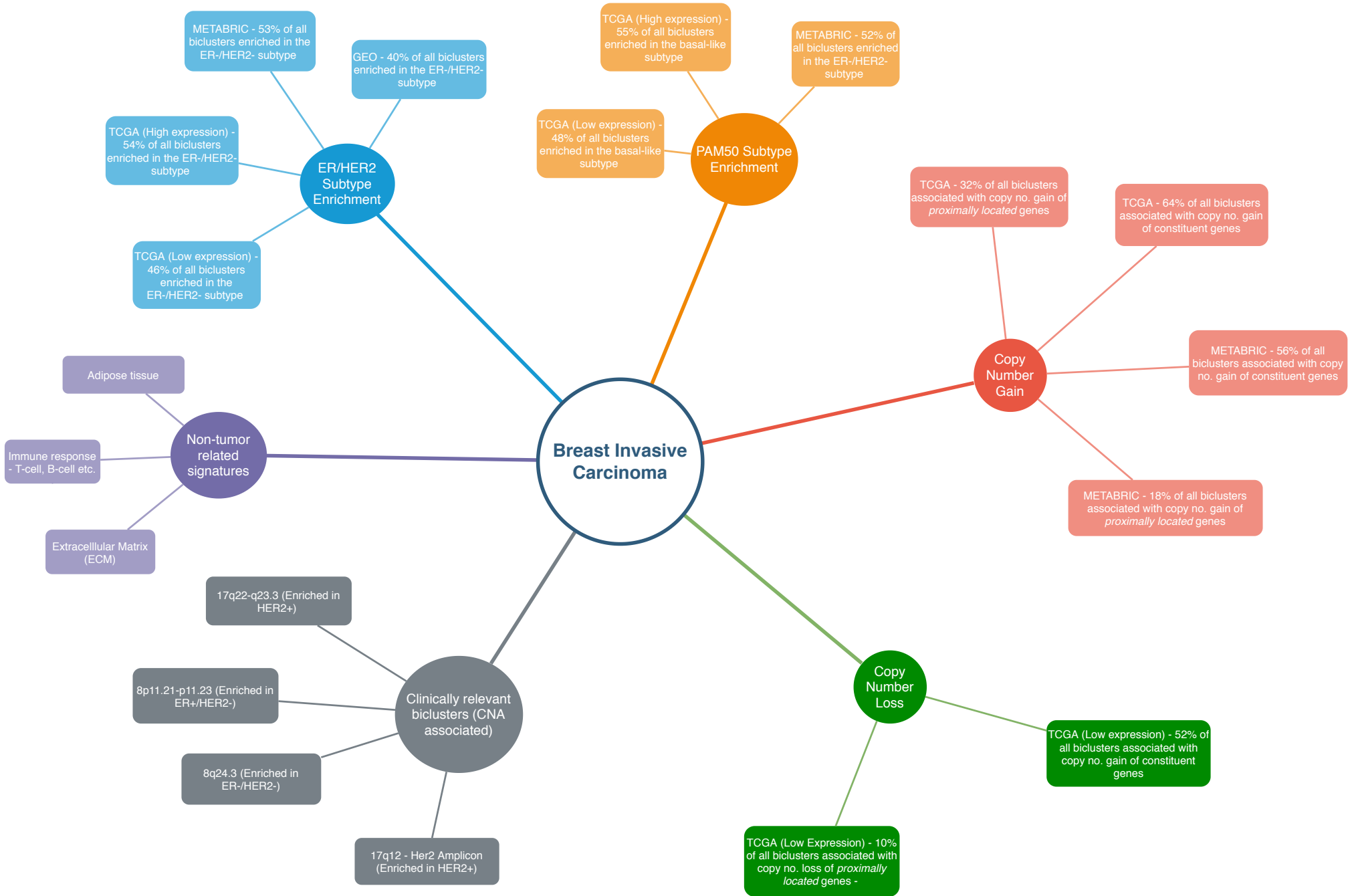

Supplement: giz064_Supplemental_Files [file giz064_supplemental_files.zip › Singh_etal.SupplementaryFigures.pdf]
