# Supplementary material for: TuBA: Tunable biclustering algorithm reveals clinically relevant tumor transcriptional profiles in breast cancer
Source: Gigascience. 2019 Jun 18;8(6):giz064. doi: 10.1093/gigascience/giz064 (PMC6582332; doi:10.1093/gigascience/giz064)
Supplement: giz064_Response_to_Reviewer_Comments_Original_Submission [file giz064_response_to_reviewer_comments_original_submission.pdf]

Author's Response To Reviewer Comments

Close

Dear Dr. Zhou:

Please find enclosed the revised version of our manuscript entitled “TuBA: Tunable Biclustering Algorithm Reveals Clinically Relevant Tumor Transcriptional Profiles in Breast Cancer” (No. GIGA-D-19-00050). We are excited about the potential acceptance of the paper and thank you and the reviewers for your positive and constructive comments. In this revision, we have addressed the comments by reviewer 3 and provide a point-by-point response below to these comments. We also have discussed the application of TuBA to large transcriptomic datasets independent of the platform for data acquisition. We agree that addressing these comments have strengthened the manuscript, and we hope that you find this revised version of our paper suitable for publication in GigaScience.

Sincerely,

Hossein Khiabanian

Reviewer 3: In this paper the authors present a tunable biclustering algorithm (TuBA), based in the use of a proximity measure. TuBA has been applied to Breast Cancer Analysis. The paper is well organized and the contents include an extensive analysis on different datasets. Nevertheless, I have the following concerns regarding its appropriateness for being accepted.

Response: We thank the reviewer for their positive comments and hope that in the revised manuscript, we have clarified and addressed these concerns.

Reviewer 3: In section METHODS, subsection "Proximity Measure" does not provide enough details in order to reproduce its computation. It is not easy to understand how it does evaluate biclusters.

Response: TuBA’s proximity measure is based on computing the significance of the overlap between the samples that exhibit high (or low) expression of any given gene pair. As noted in lines 110-114, “The number of samples shared between any pair of percentile sets follows the hypergeometric distribution; therefore, we can compute the significance (p-value) of overlaps between pairs of percentile sets based on the numbers of shared samples by using the one-sided Fisher's exact test.” The lower the p-value of overlap, the stronger is the association between the gene-pair. We illustrate the basic idea and the setup of the test underlying our proximity measure in Figure 1.

Reviewer 3: In section METHODS, subsection "Datasets" it is not clear how many datasets have been finally analysed. Although in line 248 it is said that "we applied TuBA to three BRCA datasets", TCGA, METABRIC and GEO, from line 258 some other datasets are introduced: Bicmix or GTEx.

Response: The primary and clinically relevant results of the paper are based on the application of TuBA to three large breast cancer (BRCA) datasets – TCGA, METABRIC, and GEO. We additionally used the Genotype-Tissue Expression (GTEx) and ESTIMATE datasets to reaffirm that some of our biclusters from the three BRCA datasets were associated with normal/stromal tissue gene expression signatures. Moreover, for comparing TuBA with the Bicmix algorithm, we applied TuBA to the breastCancerNKI dataset. We have now separated this subsection in Methods to clearly indicate primary and validation datasets (lines 248-289).

Reviewer 3: In section RESULTS, subsection "Benchmarking TuBA's Proximity Measure", proximity measure has been checked against correlation coefficients such as Pearson or Spearman, but not against other evaluation measures specific for biclusters.

Response: TuBA’s proximity measure, like Pearson’s and Spearman’s correlation coefficients, is a

pairwise measure of proximity. It enables us to identify gene-pairs that show strong association by virtue of a significant overlap in their top (or bottom) samples. Such gene-pairs are represented graphically as a pair of nodes linked together; the link represents the set of samples that are shared in their respective top (or bottom) percentile sets. Thus, our proximity measure provides the basis for the generation of graphs that are subsequently analyzed by our iterative graph-based approach to identify biclusters. By itself, the proximity measure does not evaluate biclusters.

We should note that TuBA does not involve modeling of gene expression data in order to identify biclusters. Thus, bicluster evaluation measures based on models of patterns of gene expression data are not applicable to its results. However, in the Enrichment of bicluster samples in top (bottom) sample sets of bicluster genes subsection of Results, we are now proposing a simple metric to assess the overall quality of the biclusters discovered by TuBA (Lines 404-412): "… the FDR values for each gene (sample) in any given bicluster can be viewed as their scores – the closer the value of the FDR is to zero for a gene (sample), the stronger is the association of the gene (sample) to the bicluster. We used the FDR values for the genes and samples within bicluster i to evaluate its overall quality, Q(Bi), defined as the minimum of the fraction of the genes in the bicluster with FDR < 0.05 or the fraction of samples in the bicluster with FDR < 0.05. Q(Bi) takes values between 0 and 1, where values close to zero indicate weak associations between the constituent genes and samples within the bicluster, while values close to one would indicate strong associations." Supplementary Table 5 is now updated to include these quality scores for the biclusters.

Reviewer 3: In section RESULTS, subsection "Choice of parameters for TuBA" it not specified a clear strategy for choosing the parameter values when analysing a new dataset.

Response: We have discussed the considerations that underlie the choices for TuBA's parameters in the Tuning TuBA subsection of Methods, also illustrated in Figure 3. For the choice of the second parameter (Overlap significance cutoff), we proposed the following heuristic in the same section: "the cutoff for the significance level of overlap should be such that a decrease in the significance level by an order of magnitude leads to an 40-60% increase in the number of edges that get added to the graph." (Lines 233-237)

Reviewer 3: Furthermore, being the results section so large, I also suggest to include a summary table or diagram in order to provide the reader with a quick snapshot of the results.

Response: We have summarized the key results and discussions in Supplementary Figure 10.

Close
