# Supplementary material for: TuBA: Tunable biclustering algorithm reveals clinically relevant tumor transcriptional profiles in breast cancer
Source: Gigascience. 2019 Jun 18;8(6):giz064. doi: 10.1093/gigascience/giz064 (PMC6582332; doi:10.1093/gigascience/giz064)
Supplement: giz064_Reviewer_1_Report_Original_Submission — JiÅ™ÃKlÃ©ma -- 4/2/2019 Reviewed [file giz064_reviewer_1_report_original_submission.pdf]

Reviewer Report

Title: TuBA: Tunable Biclustering Algorithm Reveals Clinically Relevant Tumor Transcriptional Profiles in Breast Cancer

Version: Original Submission **Date:** 4/2/2019

Reviewer name: JiÅ™Ã- KlÃ©ma

Reviewer Comments to Author:

The authors propose a new biclustering method and demonstrate its applicability in a set of breast invasive carcinoma cohorts. The method stems from the idea of gene proximity based on mutual agreement in the sets of samples where genes from a gene pair are expressed highest or lowest. Based on proximity, a gene graph is constructed, biclusters correspond to densely connected genes and samples overrepresented on the subgraph edges. The idea of gene similarity defined by the size and significance of the overlaps between the sample sets that fall into percentile sets of the inidvidual genes is plausible and appealing.
This manuscript is a resubmission of the previous paper that I also reviewed. I checked the changes that the authors made and verified how the improvements answer my earlier comments:
1. I had a conceptual comment to the algorithm design. I proposed to present the algorithm rather as a regular clustering with a new gene proximity measure. The authors keep their algorithm in the class of biclustering methods, however, they introduced a new experimental section named Enrichment of bicluster samples in top (bottom) sample sets of bicluster genes in which they showed that their algorithm tends to generate biclusters with core functional signatures, i.e., it is possible to identify core sample that comprise the bicluster and whose occurrence is statistically significantly enriched in the top and bottom sample sets of nearly each gene in the bicluster. This experiment sufficiently answers my doubt that genes appearing in a densely connected subgraph do not have to be co-expressed in a similar set of samples.
2. There are some principal limitations in the algorithm design. The algorithm includes the largest clique identification problem which is computationally difficult. I proposed to study runtime of the algorithm in a more detail. The authors did so in a new section named Runtime analysis. The section confirmed the expectation that TuBA has longer runtime than most of the other biclustering algorithms. Despite of the inconvenient asymptotic bounds, the authors demonstrated that the algorithm can be applied to current gene expression datasets with tens of thousands genes and thousands of samples. The runtime can be influenced by the setting of the significance level of overplap between percentile sets
3. The authors answered a few detailed technical comments that referred to Figure 2 and the Graph based algorithm.

Level of Interest

Please indicate how interesting you found the manuscript: Choose an item.

Quality of Written English

Please indicate the quality of language in the manuscript: Choose an item.

Declaration of Competing Interests

Please complete a declaration of competing interests, considering the following questions:

- Have you in the past five years received reimbursements, fees, funding, or salary from an organisation that may in any way gain or lose financially from the publication of this manuscript, either now or in the future?
- Do you hold any stocks or shares in an organisation that may in any way gain or lose financially from the publication of this manuscript, either now or in the future?
- Do you hold or are you currently applying for any patents relating to the content of the manuscript?
- Have you received reimbursements, fees, funding, or salary from an organization that holds or has applied for patents relating to the content of the manuscript?
- Do you have any other financial competing interests?
- Do you have any non-financial competing interests in relation to this paper?

If you can answer no to all of the above, write 'I declare that I have no competing interests' below. If your reply is yes to any, please give details below.

I declare that I have no competing interests.

I agree to the open peer review policy of the journal. I understand that my name will be included on my report to the authors and, if the manuscript is accepted for publication, my named report including any attachments I upload will be posted on the website along with the authors' responses. I agree for my report to be made available under an Open Access Creative Commons CC-BY license (http://creativecommons.org/licenses/by/4.0/). I understand that any comments which I do not wish to be included in my named report can be included as confidential comments to the editors, which will not be published.

I agree to the open peer review policy of the journal

To further support our reviewers, we have joined with Publons, where you can gain additional credit to further highlight your hard work (see: https://publons.com/journal/530/gigascience). On publication of this paper, your review will be automatically added to Publons, you can then choose whether or not to claim your Publons credit. I understand this statement.

Yes Choose an item.
