# Supplementary material for: TuBA: Tunable biclustering algorithm reveals clinically relevant tumor transcriptional profiles in breast cancer
Source: Gigascience. 2019 Jun 18;8(6):giz064. doi: 10.1093/gigascience/giz064 (PMC6582332; doi:10.1093/gigascience/giz064)
Supplement: giz064_Reviewer_3_Report_Original_Submission — Beatriz Pontes -- 4/4/2019 Reviewed [file giz064_reviewer_3_report_original_submission.pdf]

Reviewer Report

Title: TuBA: Tunable Biclustering Algorithm Reveals Clinically Relevant Tumor Transcriptional Profiles in Breast Cancer

Version: Original Submission **Date:** 4/4/2019

Reviewer name: Beatriz Pontes

Reviewer Comments to Author:

In this paper the authors present a tunable biclustering algorithm (TuBA), based in the use of a proximity measure. TuBA has been applied to Breast Cancer Analysis.
The paper is well organized and the contents include an extensive analysis on different datasets. Nevertheless, I have the following concerns regarding its appropriateness for being accepted:
- In section METHODS, subsection "Proximity Measure" does not provide enough details in order to reproduce its computation. It is not easy to understand how it does evaluate biclusters.
- In section METHODS, subsection "Datasets" it is not clear how many datasets have been finally analysed. Although in line 248 it is said that "we applied TuBA to three BRC datasets", TCGA, METABRIC and GEO, from line 258 some other datasets are introduced: Bicmix or GTEx.
- In section RESULTS, subsection "Benchmarkig TuBA's Proximity Measure", proximity measure has been checked against correlation coefficients such as Pearson or Spearman, but not against other evaluation measures specific for biclusters.
- In section RESULTS, subsection "Choice of parameters for TuBA" it not specified a clear strategy for choosing the parameter values when analysing a new dataset.
Furthermore, being the results section so large, I also suggest to include a summary table or diagram in order to provide the reader with a quick snapshot of the results.

Level of Interest

Please indicate how interesting you found the manuscript: Choose an item.

Quality of Written English

Please indicate the quality of language in the manuscript: Choose an item.

Declaration of Competing Interests

Please complete a declaration of competing interests, considering the following questions:

- Have you in the past five years received reimbursements, fees, funding, or salary from an organisation that may in any way gain or lose financially from the publication of this manuscript, either now or in the future?

- Do you hold any stocks or shares in an organisation that may in any way gain or lose financially from the publication of this manuscript, either now or in the future?
- Do you hold or are you currently applying for any patents relating to the content of the manuscript?
- Have you received reimbursements, fees, funding, or salary from an organization that holds or has applied for patents relating to the content of the manuscript?
- Do you have any other financial competing interests?
- Do you have any non-financial competing interests in relation to this paper?

If you can answer no to all of the above, write 'I declare that I have no competing interests' below. If your reply is yes to any, please give details below.

I declare that I have no competing interests

I agree to the open peer review policy of the journal. I understand that my name will be included on my report to the authors and, if the manuscript is accepted for publication, my named report including any attachments I upload will be posted on the website along with the authors' responses. I agree for my report to be made available under an Open Access Creative Commons CC-BY license (http://creativecommons.org/licenses/by/4.0/). I understand that any comments which I do not wish to be included in my named report can be included as confidential comments to the editors, which will not be published.

I agree to the open peer review policy of the journal

To further support our reviewers, we have joined with Publons, where you can gain additional credit to further highlight your hard work (see: https://publons.com/journal/530/gigascience). On publication of this paper, your review will be automatically added to Publons, you can then choose whether or not to claim your Publons credit. I understand this statement.

Yes Choose an item.
